# Effect of Load Spatial Configuration on the Heating of Chicken Meat Assisted by Radio Frequency at 40.68 MHz

**DOI:** 10.3390/foods11081096

**Published:** 2022-04-11

**Authors:** Sandro M. Goñi, Matteo d’Amore, Marta Della Valle, Daniela F. Olivera, Viviana O. Salvadori, Francesco Marra

**Affiliations:** 1 Centro de Investigación y Desarrollo en Criotecnología de Alimentos (CIDCA), Conicet La Plata-Universidad Nacional de La Plata-Comisión de Investigaciones Científicas, 47 y 116, La Plata 1900, Argentina; smgoni@quimica.unlp.edu.ar (S.M.G.); danielaolivera@conicet.gov.ar (D.F.O.); vosalvad@ing.unlp.edu.ar (V.O.S.); 2 Facultad de Ingeniería, Universidad Nacional de La Plata, 1 y 47, La Plata 1900, Argentina; 3 Dipartimento di Farmacia, Università degli Studi di Salerno, 84084 Fisciano, Italy; mdamore@unisa.it; 4 Dipartimento di Ingegneria Industriale, Università degli Studi di Salerno, 84084 Fisciano, Italy; m.dellavalle1@studenti.unisa.it; 5 Facultad de Ciencias Veterinarias, Universidad Nacional de La Plata, 60 y 118, La Plata 1900, Argentina

**Keywords:** radiofrequency, heating, energy efficiency, digital twin

## Abstract

Food heating assisted by radio frequencies has been industrially applied to post-harvest treatment of grains, legumes and various kind of nuts, to tempering and thawing of meat and fish products and to post-baking of biscuits. The design of food processes based on the application of radiofrequencies was often based on rules of thumb, so much so that their intensification could lead significant improvements. One of the subjects under consideration is the shape of the food items that may influence their heating assisted by radiofrequency. In this work, a joint experimental and numerical study on the effects of the spatial configuration of a food sample (chicken meat shaped as a parallelepiped) on the heating pattern in a custom RF oven (40.68 MHz, 50 Ohm, 10 cm electrodes gap, 300 W) is presented. Minced chicken breast samples were shaped as cubes (4 × 4 × 4 cm^3^) to be organized in different loads and spatial configurations (horizontal or vertical arrays of 2 to 16 cubes). The samples were heated at two radiofrequency operative power levels (225 W and 300 W). Heating rate, temperature uniformity and heating efficiency were determined during each run. A digital twin of the experimental system and process was developed by building and numerically solving a 3D transient mathematical model, taking into account electromagnetic field distribution in air and samples and heat transfer in the food samples. Once validated, the digital tool was used to analyze the heating behavior of the samples, focusing on the most efficient configurations. Both experiments and simulations showed that, given a fixed gap between the electrodes (10 cm), the vertically oriented samples exhibited a larger heating efficiency with respect to the horizontally oriented ones, pointing out that the gap between the top electrode and the samples plays a major role in the heating efficiency. The efficiency was larger (double or even more; >40% vs. 10–15%) in thicker samples (built with two layers of cubes), closer to the top electrode, independently from nominal power. Nevertheless, temperature uniformity in vertical configurations was poorer (6–7 °C) than in horizontal ones (3 °C).

## 1. Introduction

In dielectric heating (which can be referred to both radio frequency—RF—and microwaves—MW) heat is volumetrically generated inside a sample (often simply called load) due to the interaction of the electromagnetic field the load is placed in, with the ionic and polar molecules contained in the sample. In traditional heating, heat is transferred from the surrounding medium to the sample by convection and conduction mechanisms, the latter being particularly slow in foods due to their low thermal conductivity. Dielectric heating provides higher heating rates and reduces processing times with respect to processes controlled by heat conduction or convection [1]. 

Guo et al. [2] reviewed the recent literature on RF heating applied to fresh food processing. The benefits of RF, basically due to rapid heating, deep thermal penetration and possibility of quality control, were investigated in different processes, such as cooking [3,4], post-harvest treatment of agriculture commodities [5,6], pasteurization [7,8], drying [9], tempering [10] and thawing [11,12,13].

Dielectric heating in the RF range is generally performed at three defined frequencies, i.e., 13.56 MHz, 27.12 MHz and 40.68 MHz, even if most of the studies reported in the scientific literature refer to RF-assisted processes at 27.12 MHz. In RF cooking of different meats, Laycock et al. [14] investigated the effect of RF on quality, heating rate and temperature profiles of three types of meat products (ground, comminuted and muscle) cooked in a 1.5 kW, 27.12 MHz RF heater. Authors concluded that ground and comminuted products seem to be promising for RF cooking, as cooking times decreased by more than 80%. Kirmaci and Singh [15] compared RF with water bath (WB) cooking of fresh and marinated chicken breast meat, using a 6 kW, 27.12 MHz RF oven. RF cooking resulted in a higher heating rate, achieving cooking times that were 42% shorter. Similar quality parameters were measured in both procedures, although RF cooked meat had lower redness. Muñoz et al. [16] analyzed a two-step cooking process for pork hams, which included a RF tunnel and a steam oven, and compared the results with hams completely processed in the steam oven. Authors pointed out that cooking time decreased by 50% using RF, with no significant differences observed in the terms of the sensory quality of the final products. Wang et al. [17] applied RF energy at 27.12 MHz to pork tenderloin, demonstrating that RF heating improved water retention of pork myofibrillar protein gel and it had the potential to improve meat quality and could greatly reduce the processing time, furthermore facilitating the formation of a stable and orderly gel network structure.

Although RF heating is claimed to be a method to obtain uniform temperature distribution in the heated product, a common issue of RF heating is the lack of temperature uniformity, which produces zones or spots of high and low temperatures inside the heated food. The temperature uniformity depends on the way electromagnetic energy is spatially absorbed by food, which has, in turn, a complex dependence on several factors [18]. A paramount factor affecting heating uniformity is the deflection of the electric field because of the sample, which concentrates the net electric field in certain regions that are heated quicker [19,20]. Other factors can be grouped into three classes: food characteristics, such as dielectric and thermal properties, geometry, size and mass; RF system features such as power, electrodes shape and size, gap between electrodes; and factors related to the RF application, such as food orientation on the cavity, position of the sample between the electrodes, manipulation of electrode positions (for adjustable gap devices), food area related to electrode area, and the use of special food containers [18]. Different strategies to improve heating uniformity have been extensively reviewed by [18]. Among others, Romano and Marra [21] used modeling and simulation to analyze the effects of regular geometries (a cube, cylinder and sphere of equal volume) and orientation on temperature uniformity during heating at 27.12 MHz, 400 W maximum power, using properties of luncheon meat batter. The cubic shape presented the best temperature uniformity and the highest heating rate, followed by the vertical cylinder. The sphere and horizontal cylinder had lower heating rates and worse temperature uniformity. Recently, Li et al. [22] discussed a strategy for improving the uniformity of radio frequency tempering for frozen beef with cuboid and step shapes; Bedane et al. [23] experimentally investigated the same aspects using regular geometries of equal volume (a cube, cylinder and sphere) of tylose with different salt concentrations. A 27.12 MHz, 10 cm electrode gap, 600 W maximum power RF cavity was employed, and the samples were heated for 6 min. The temperature was measured at different locations. The vertical cylinder and cube presented the best temperature uniformity, while the sphere and horizontal cylinder had the worst one. Both studies clearly demonstrate the effect of geometry and orientation on RF heating performance. Tiwari et al. [20] simulated the heating of dry foods in a 27.12 MHz, 12 kW RF system. Several simulations varying the geometry (cuboids, cylinders and ellipsoids), the size and position of cuboid-shaped samples, the gap between electrodes, etc., were performed. A power uniformity index (PUI) was easily obtained from the numerical results. The best (lowest) PUI was obtained for ellipsoids in the middle of the oven, followed by the cylinder and cuboid. Theoretical predictions show that for cuboids the uniformity can be improved increasing the sample size. Additionally, for different cuboid sizes, different vertical positions led to different optimal PUI locations. Later, Tiwari et al. [24] successfully validated the previous model through experiments using hard red spring wheat flour. In cooking of fresh and marinated chicken breast, Kirmaci and Singh [15] obtained a better temperature uniformity with water bath cooking (0.9 °C) with respect to RF (5.3 °C), even if in the water bath a longer time was required. Uyar et al. [3] used a simulation to analyze the effect of volume of cubic meat samples on heating rate and efficiency at 27.12 MHz. Two cases were considered: a fixed electrode gap and a fixed electrode-sample distance (air gap). Results showed the great impact of the sample load and air gap on the heating rate and efficiency. For a fixed electrode gap, the heating rate and efficiency improved with the sample volume increase. For a fixed air gap, the heating rate was higher for small loads, but the efficiency was higher for large loads. Information about temperature uniformity was not reported. In a further study, Uyar et al. [4] simulated the effect of the projected area during heating of meat samples on several uniformity indexes, such as PUI. It was found that configurations leading to a higher temperature increase also have a less uniform temperature distribution. So, this opposite behavior between desirable objectives led to the need for an increase in the studies in the design of the RF cavity [18]. 

In summary, the influence of geometric characteristics on power absorption uniformity is proven. Notwithstanding, there is a lack of studies oriented to the heating (cooking) of fresh products. Besides, the applicability of different RF frequencies, such as 40.68 MHz, which is more appropriate for heating small volume pieces due to its smaller penetration depth, has not been sufficiently investigated [2]. Therefore, the objective of this work was to study the effect of load spatial configuration on heating rate, temperature uniformity and energy efficiency during RF heating of chicken meat at 40.68 MHz. 

## 2. Materials and Methods

### 2.1. Food Samples

Minced lean chicken breast bought at a local butcher shop in Salerno, Italy, was used in the experimental heating tests. Sixteen plastic cubic containers (4 × 4 × 4 cm^3^) were fabricated with an advanced desktop 3D printer, Replicator 2X Experimental 3D (MakerBot Industries, Brooklyn, NY, USA), with dual extrusion to print with filaments. Acrylonitrile butadiene styrene (ABS), a thermoplastic polymer characterized by a high impact resistance and toughness, and electrical properties that are constant over the RF range [25] were used to print the containers.

**Figure 1:**
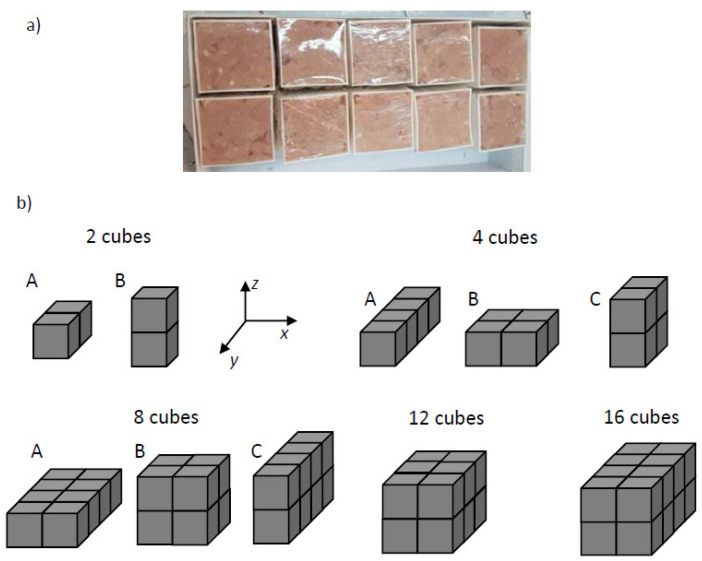
(**a**) Minced breast meat into the cubic containers, sealed with plastic film; (**b**) spatial configuration of the different samples used in the heating tests.

Each mold was filled with 60 g of minced chicken breast, paying attention to filling all the mold’s volume, and then wrapped with plastic film as shown in Figure 1a. Before each heating test, the samples were equilibrated in a refrigerator at 277 K.

### 2.2. RF Oven

An experimental prototype of RF oven (40.68 MHz, 50 Ohm, 300 W maximum power) was used to perform the heating tests. The RF oven consisted of an electrically insulated chamber 275 mm width, 304 mm depth, 170 mm height, with a front door of 260 mm × 95 mm, a RF generator, a RF amplifier and a RF applicator (280 mm × 240 mm fixed parallel rectangular electrodes, 10 mm gap). The RF power is controlled by a matching system provided by the RF cavity manufacturer, which adjusts the impedance and the resistivity of the whole circuit to make it resonate at the operating frequency. The whole system is remotely controlled by an Android app [26]. Many details of the RF prototype cannot be revealed due to an ongoing non-disclosure agreement with the manufacturer.

### 2.3. Heating Tests

Two power levels were used during the heating experiments: the maximum power of the prototype, i.e., 300 W, and 225 W. Complete details of the experiments are given in Table 1: spatial configuration, sample size, area projected over the electrodes, sample mass and heating times. All experiments were run in five replicates.

Ten sample configurations (formed by sets of 2 to 16 meat cubes) were tested in each set. The cubic shape is used as a reference geometry as this geometry exhibits a more uniform heating rate and power absorption [21,23]. The cubes were arranged as samples of different sizes, all of them exposing a planar surface to the electrodes in the RF cavity. Figure 1b shows the geometric configuration of the samples as they were placed inside the cavity. The area projected over the electrodes is the sample area crossed by the electric field formed between the two electrodes [4].

Experimental heating times (also reported in Table 1) were defined to avoid reaching the saturation temperature, i.e., 100 °C, at atmospheric pressure. This condition also allows us to ignore the heat losses due to moisture transfer.

Optical fibers (TS2, Optocon, Weidmann Technologies Deutschland GMBH, Dresden, Germany) were used to measure the sample temperature, every 10 s, at characteristic points: the center and one or more corners of each cube; thus providing several transient values of temperature profiles for each sample. 

The experiments allowed us to evaluate the influence of the sample mass and spatial configuration on the heating performance of the oven, measured in terms of heating rate (referred to the heating time), temperature uniformity and energy efficiency. 

The heating rate *HR* (in K s^−1^) was defined in as:

(1) $$HR=\frac{T-T_{0}}{t_{h}}$$

where *T* is the sample temperature (K), *T*_0_ is the initial sample temperature (K) and *t_h_* is the heating time (s). *HR* was evaluated for a single point of the cube, for a cube (averaging the *HR* for all measured points), or for a sample (averaging *HR* for all cubes constituting the sample). 

The experimental average temperature increase *T_Inc,Exp_* (Equation (2)) was calculated as the difference between the average final temperature *T_Ave,Exp_* (Equation (3)) and the initial temperature $T_{0}$.

(2) $$T_{Inc,Exp}=T_{Ave,Exp}-T_{0}$$

(3) $$T_{Ave,Exp}=\frac{\sum_{i=1}^{N}T_{i}}{N}$$

where *N* is the number of points where the temperature has been measured.

Additionally, the experimental temperature uniformity *T_U,Exp_* was assessed as the absolute temperature deviation of the temperature of each point *T_i_* from the average temperature:

(4) $$T_{U,Exp}=\frac{\sum_{i=1}^{N}\left|T_{i}-T_{Ave,Exp}\right|}{N}$$

Energy efficiency measures the quantity of energy absorbed by the sample, with reference to the energy supplied by the RF oven, i.e., the nominal value. According to the experimental value of final average temperature *T_Ave,Exp_*, the averaged absorbed energy was calculated as:

(5) $$Q_{Exp}=m\;C_{P}\;\left(T_{Ave,Exp}-T_{0}\right)$$

where *m* is the sample mass (kg), *C_P_* is the specific heat capacity (J kg^−1^ K^−1^), while experimental energy efficiency $\eta_{Exp}\left(\%\right)$ was defined as:

(6) $$\eta_{Exp}\left(\%\right)=100\frac{Q_{Exp}}{\left(t_{h}\;NP\right)}$$

where *t_h_* is the experimental heating time (s), and *NP* is the nominal power (W), then the term ($t_{h\;}NP)$ (in J) is the nominal energy provided by the RF oven. 

### 2.4. RF Heating Model

Dielectric heating is a complex multi-physics problem where heat transfer and electric field displacement inside and around the food sample must be considered simultaneously, and the two phenomena must be described and solved together. The phenomena are strictly interconnected: as a matter of fact, while the electric field distribution is affected by the sample dielectric properties (which, in turn, are strongly dependent on the temperature and the food composition), the heat balance within the food sample depends on the electromagnetic heat source, which in turn depends on the local modulus of the electric field and on food dielectric properties. In order to solve the coupled system, a specialized frequency-transient solver in COMSOL^TM^ Multiphysics was employed (COMSOL AB, Sweden [27]). In the built model, the COMSOL AC/DC module (which provides mathematical description for the electromagnetic field in the RF range), and the transient heat transfer module were used. 

**Figure 2:**
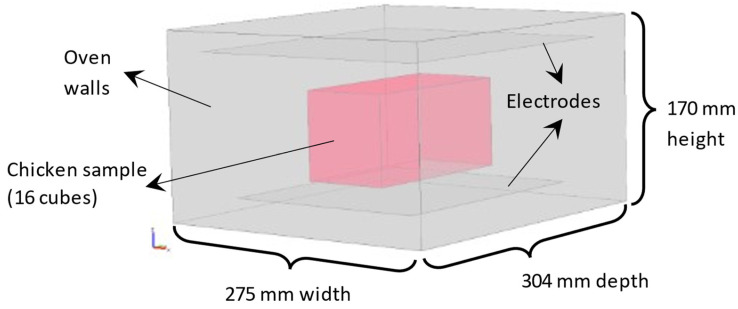
Scheme of the RF oven and a food sample used in simulations.

A tridimensional scheme of the system was built (Figure 2). The distance between the sample and the bottom electrode was 0.015 m; the distance between the sample and the top electrode depends on sample configuration. Samples sizes and configurations detailed in Table 1 were simulated. 

#### 2.4.1. Governing Equations

Heat transfer balance was solved only in the food sample, imposing boundary conditions at the interfaces between sample and surrounding environment, while the electromagnetic field distribution was solved in both air and food domains. 

The transient heat transfer inside the food sample was described according to Equation (7):

(7) $$\rho C_{P}\frac{\partial T}{\partial t}=\nabla \cdot \left(k\nabla T\right)+\dot{Q}$$

where *ρ* is the density (kg m^−3^), *C_P_* the specific heat capacity (J kg^−1^ K^−1^) and *k* the thermal conductivity (W m^−1^ K^−1^) of the food sample. The electromagnetic heat source $\dot{Q}$ (W m^−3^) was defined as (Huang et al., 2018):

(8) $$\dot{Q}=2\pi \omega \varepsilon_{0}\varepsilon^{\prime \prime }{\left|E\right|}^{2}$$

where ω is the electric field frequency (Hz), *ε*_0_ is the permittivity of free space (8.85 × 10^−12^ Fm^−1^), *ε*″ is the dielectric loss factor of the sample and |*E*| (V m^−1^) is the modulus of the electric field vector. 

Initial uniform condition for temperature was considered (277 K, experimental data), while convective heat transfer boundary condition between air and sample surface was modeled using a heat transfer coefficient (*h* = 10 W m^−2^ K^−1^, *T_air_* = 293 K):

(9) $$-k\nabla T=h\left(T-T_{air}\right)$$

The RF wavelength (about 7.37 m) at the employed frequency was larger than the electrode gap, so the Maxwell’s equations reduce to Equation (10), which was used to model the displacement of the electric field [3,19,24,28]:

(10) ∇·εE=0

where *ε* is the relative complex permittivity of the material (F m^−1^), which depends on the dielectric constant *ε*′ and the dielectric loss factor *ε*″ of the material (*ε = ε′ – jε″*). It should be noted that although the electric field model is steady, it is solved at each time step of the heat transfer transient simulation.

Boundary conditions to determine the electric field distribution inside the RF oven were set up as:

- Bottom electrode was maintained at the ground condition (*V* = 0);
- Top electrode was maintained at a constant potential *V*_0_ according with the applied output power (300 or 225 W) with a frequency of 40.68 MHz;
- Oven walls were electrically insulated, ∇·E=0.

The chicken breast thermophysical properties were estimated from its composition and temperature *T*, according to the relationships proposed by [29]. In general, the lipid content of chicken breast meat is lower than 1.2% [30]; therefore, for the purpose of this work, the composition was simplified as 75% water and 25% protein. Then, the resulting thermophysical properties were expressed as:

(11) $$\rho =-3.087\;10^{-3}T^{2}+1.597\;T+839.5$$

(12) $$C_{P}=3.602\;10^{-3}T^{2}-1.721\;T+3834$$

(13) $$k=-5.579\;10^{-6}\;T^{2}+4.471\;10^{-3}T-0.3319$$

As was described in Equation (8), the dielectric properties characterize the absorption of incident RF power. In this work, chicken dielectric properties were estimated from experimental data measured by [31], who measured the dielectric constant $\varepsilon^{\prime }$ and the dielectric loss factor $\varepsilon^{\prime \prime }$ of uncooked chicken breast meat at 51 different frequencies, in the range from 10 MHz to 1.8 GHz. Additionally, the influence of temperature on dielectric properties was recorded, with measures in the range from 5 to 85 °C. 

**Figure 3:**
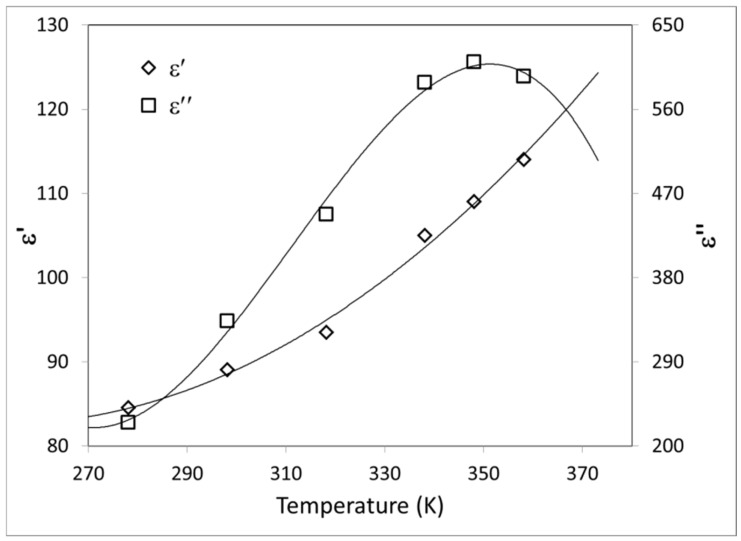
Estimated dielectric properties of chicken breast. Dielectric constant ε′ (◇) and loss factor ε″ (☐) vs. temperature, at 40.68 MHz. The lines are the fitting of Equations (14) and (15).

To adequately represent the dielectric properties of chicken breast meat in the numerical model, the values reported by [31] at 40 MHz and different temperatures were fitted. Average dielectric properties of pectoralis minor and major muscles were employed in the fitting procedure. Figure 3 shows the dependence of both dielectric properties on temperature *T*. The dielectric constant monotonically increases with temperature from 278 to 358 K (5 to 85 °C). This behavior is also observed at other frequencies. Instead, the loss factor increases until it reaches a maximum, which depends on the frequency; at 40 MHz the maximum is observed near to 348 K. Equations (14) and (15) represent the fitted equations used in the model:

(14) $$\varepsilon^{\prime }=2.865\;10^{-3}T^{2}-1.4469\;T+265.23$$

(15) $$\varepsilon^{\prime \prime }=-1.4042\;\;10^{-3}T^{3}+1.3107\;T^{2}-400.7\;T+40499$$

For air, the dielectric constant *ε*′ is 1, whereas dielectric loss factor *ε*″ is 0. 

#### 2.4.2. Numerical Solution

We used a custom computer equipped with 8 CPUs Intel I7-3820 @ 3600 GHz FSB (front side bus, Intel, Santa Clara, CA, USA); motherboard MSI X79A-GD65 (8D) certified for military use according to the standard MIL-STD-810G, 1.600 MHz Socket (Micro-Start Int’l Co., Ltd., New Taipei City, Taiwan); Socket R (LGA 2011, Intel, Santa Clara, CA, USA) 4 cores; equipped with a RAM of 64 Gb DDR3 1600 MHz. The workstation runs under Windows 7 Professional (Microsoft, Redmond, WA, USA) operating system at Department of Industrial Engineering, University of Salerno, Italy. Time-dependent equations were discretized using the 2^nd^-order Backward Differentiation Formula (BDF) method, with maximum time steps of 5 s; MUMPS solver was used to solve the resulting equations with relative and absolute tolerances of 0.01 and 0.1, respectively.

##### Simulated Variables

From the mathematical model, different features can be determined: among others, average temperature, average temperature increase, temperature uniformity, absorbed energy and energy efficiency.

Volume average temperature at any time was calculated as:

(16) $$T_{Ave,Sim}\left(t\right)=\frac{1}{V_{sample}}\int_{V}^{\;}TdV$$

where *V_sample_* (m^3^) is the sample volume. Average temperature increase was evaluated at any time during heating as the volumetric deviation from initial temperature *T*_0_:

(17) $$T_{Inc,Sim}\left(t\right)=\frac{1}{V_{sample}}\int_{V}^{\;}\left(T-T_{0}\right)dV$$

Temperature uniformity was defined as the absolute volumetric deviation from average temperature $T_{Ave,sim}$:

(18) $$T_{U,Sim}\left(t\right)=\frac{1}{V_{sample}}\int_{V}^{\;}\left|T-T_{Ave,Sim}\right|dV$$

To estimate the efficiency of the process using the numerical model, the energy absorbed by the sample during RF heating was calculated as:

(19) $$Q_{Sim}=\overset{t_{h}}{\underset{0}{\int }}\overset{\;}{\underset{V}{\int }}\rho C_{p}\frac{\partial T}{\partial t}dVdt$$

The simulated energy efficiency $\eta_{Sim}\left(\%\right)$ was defined in Equation (20) as:

(20) $$\eta_{Sim}\left(\%\right)=100\frac{Q_{Sim}}{t_{h}\;NP}$$

Thus, the performance of the RF oven was completely characterized through the temperature uniformity and the energy efficiency. These parameters were defined analogously to the experimental variables, for comparison and validation purposes. In this sense, accuracy of the mathematical model was assessed using the percentage average absolute relative deviation (*AARD*) and average absolute deviation (*AAD*) between experimental and simulated values of average temperature (K) profiles:

(21) $$AARD\left(\%\right)=\frac{100}{n}\overset{n}{\underset{i=1}{\sum }}\left|\frac{T_{Ave,Exp,i}-T_{Ave,Sim,i}}{T_{Ave,Exp,i}}\right|$$

(22) $$AAD\left(K\right)=\frac{1}{n}\overset{n}{\underset{i=1}{\sum }}\left|T_{Ave,Exp,i}-T_{Ave,Sim,i}\right|$$

##### Mesh Independence Analysis

Mesh convergence studies were performed to ensure that the results were independent of mesh resolution. With this aim, the influence of meshing on the electric field and heat transfer solution was analyzed using the biggest sample (16 cubes). Eight default mesh configurations, from extra fine to extremely coarse, were tested. 

The volume average temperature increase (Equation (17)) and the volume average electromagnetic heat source (Equation (23)) were calculated as a function of time and used to evaluate the mesh influence.

(23) $${\dot{Q}}_{Ave}\left(t\right)=\frac{1}{V_{sample}}\int_{V}^{\;}\dot{Q}\left(t\right)dV$$

**Figure 4:**
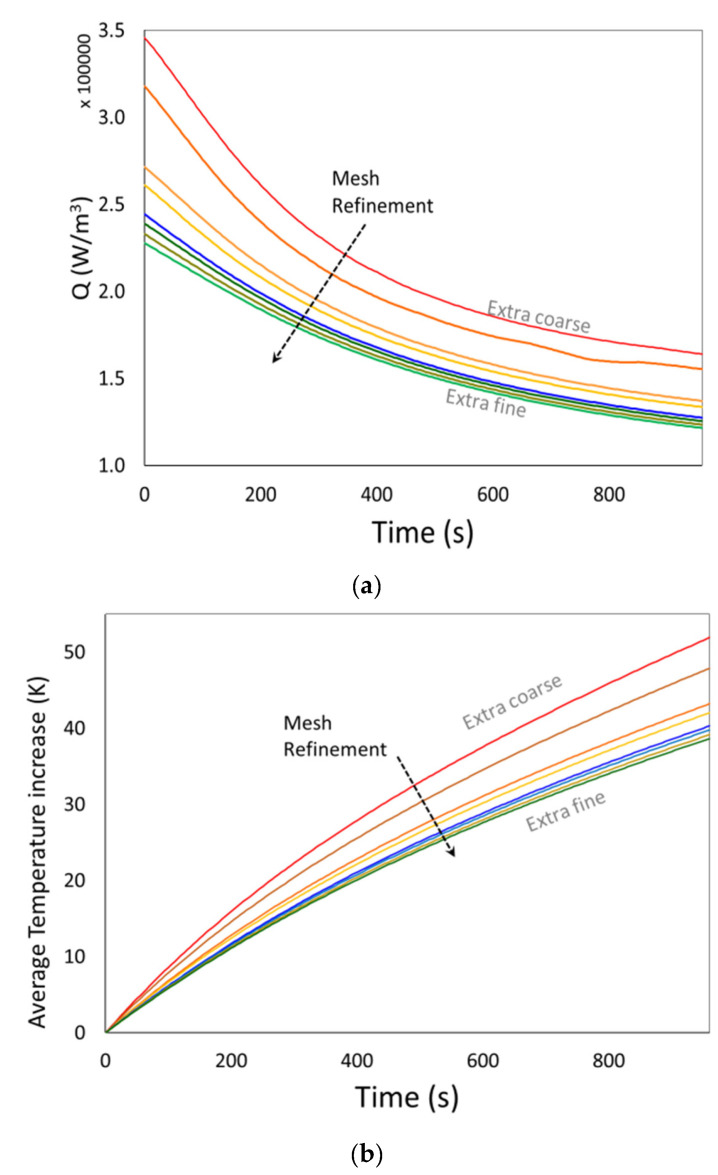
Effect of mesh refinement on simulated values, for the sample with 16 cubes, 300 W RF nominal power. (**a**) volume average electromagnetic heat source (Equation (23)) vs. time; (**b**) volume average temperature increase (Equation (17)) vs. time.

Figure 4 shows the influence of the mesh’s refinement on the average temperature increase and the average heat source versus time. Thus, the mesh size was determined based on these mesh analyses, seeking acceptable differences between successive calculations [32]. 

Table 2 reports the final average values of the mentioned quantities and the required simulations times (related to the hardware employed in this study) for the different mesh sizes. From the results of mesh analysis, the “Extra Fine” mesh was selected and employed in all cases. Different mesh analyses can be also performed, for instance setting personalized maximum and minimum finite element sizes or using different element sizes for air and chicken (due to differences in the dielectric properties of both materials). 

## 3. Results and Discussion

### 3.1. Heating Rate. Effect of Sample Mass and Spatial Configuration

The behavior of the tested sample arrays was analyzed employing the heating rate defined in Equation (1), *T* being the temperature measured at the end of heating.

The average values of *HR* for each spatial configuration, obtained in the tests with 225 and 300 W of nominal power, are presented in Table 3. 

Comparing the heating rates at different nominal power for the same configuration, it is possible to appreciate that an increase in the nominal power corresponded to an increase in the heating rate, except for the configuration 4B, which did not increase its heating rates. At a given power, it was observed that—at a given sample mass—all the configurations with the shorter possible distance from the top electrode and surface exhibited a higher heating rate. Configurations characterized by the same mass (and then by the same number of cubes) were compared: configuration 2A was compared with configuration 2B; configuration 4A with 4B and 4C; configurations 8A with 8B and 8C. 

When comparing samples made by two cubes (configurations 2A and 2B), in configuration 2B (which was vertically oriented) heating was observed to be three times faster than in the 2A one. The different orientation corresponded to a closer distance between the top electrode and the upper free surface of the sample, though the configuration 2B had a smaller projected area with respect to 2A. Uyar et al. [3] reported that a wider sample projected area together with a lower electrode gap provide higher heating rates. In the case analyzed in this work, it appeared evident that the distance between the top surface and the top electrode was the key parameter to distinguish the two cases: in configuration 2A, the sample had a greater projected area and the major gap between the top surface and the electrode, while in configuration 2B the sample had half the projected area but it was closer to the top electrode. 

In samples formed by four cubes, the highest heating rate was observed in scheme 4C (three times higher than the heating rate of samples 4A and 4B for both power levels), which again presented two rows of cubes in a vertical direction, this sample being the closest to the top electrode. Although samples 4A–4B had a greater projected area than 4C, the effect of the lower gap between sample and electrode was more relevant and dominant over the projected area.

Regarding the configuration with eight cubes, for both power levels the highest heating rate was found in the sample 8B and 8C, with two rows in a vertical direction. Samples 8B and 8C had the same projected area and the same gap between the surface and the electrode. As they were closer to the top electrode than 8A, their heating rates triplicated the heating rate of the latter. 

As the distance between the top and bottom electrode was fixed, in configurations with more than one layer of cubes the gap between the sample surface and the top electrode was smaller. Therefore, these experimental results confirmed previous theoretical results: the larger the air gap between sample and top electrode, the slower the heating rate (Uyar et al., 2014 [3]).

### 3.2. Model Validation 

**Figure 5:**
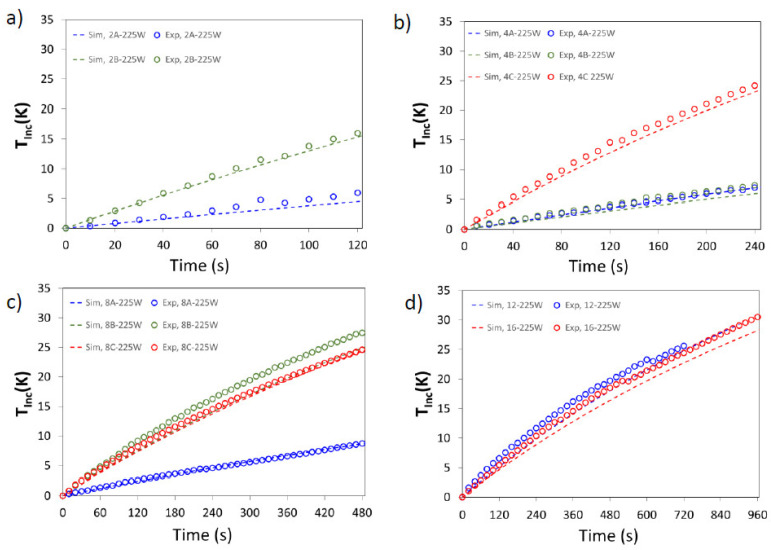
Experimental (symbols) and simulated (dashed lines) average temperature increase (*T_Inc_*, K) for 225 W. Samples: (**a**) 2 cubes; (**b**) 4 cubes; (**c**) 8 cubes; (**d**) 12 and 16 cubes.

**Figure 6:**
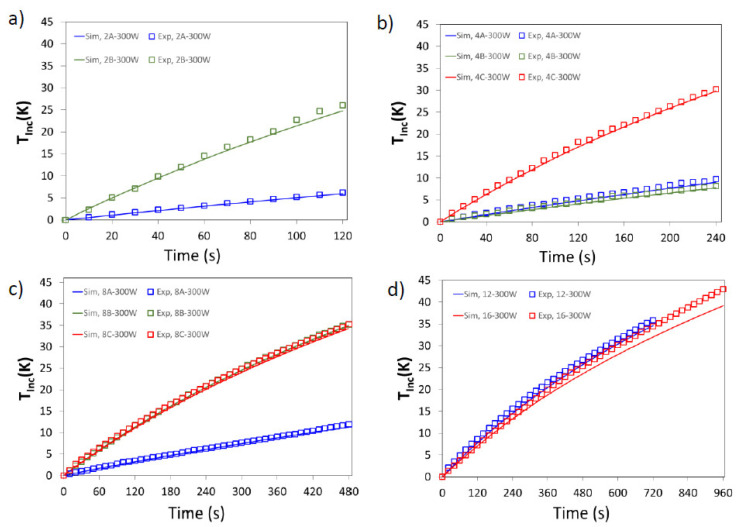
Experimental (symbols) and simulated (lines) average temperature increase (*T_Inc_*, K) for 300 W. Samples: (**a**) 2 cubes; (**b**) 4 cubes; (**c**) 8 cubes; (**d**) 12 and 16 cubes.

Experimental and simulated average temperature profiles, *T_Inc,Exp_* and *T_Inc,Sim_*, are shown in Figure 5 and Figure 6. Additionally, the accuracy of the mathematical model is evaluated by AARD and AAD, these data are summarized in Table 4.

As can be seen, the model predictions were in good agreement with experimental values; the predicted values were generally slightly lower than the experimental ones. The higher differences can be observed for sample 8B at 225 W, and 16 cubes for both powers. The numerical results were slightly lower than the experimental ones in all the considered cases, and this can be attributed to the expressions used to represent the dielectric property of the sample, taken from [31], since differences in dielectric constant and loss factor will affect the determination of EM field and the calculation of the heat generated by the interactions of the EM field with the food material, and thus the evolution of the temperature in the space and during the time.

To complete the analysis of temperature evolution, Table 5 detailed the final temperature increase, *T_Inc,Exp_* and *T_Inc,Sim_*, for both powers. Samples with the same size (and mass) presented different thermal histories and, in consequence, different average final temperatures.

The spatial configuration of the samples affected the evolution of temperature profile inside them; in those close to the top electrode, the electric field deflection is more pronounced [19,20]), increasing the heating rate and the average final temperature. Although this effect is desirable, an additional consequence is that the electric field is concentrated in the corners and edges of the sample, leading to uneven power absorption and poor temperature uniformity. Table 6 details the experimental and simulated temperature uniformity. These results indicate that samples with higher heating rates and temperature increases present worse temperature uniformity.

**Figure 7:**
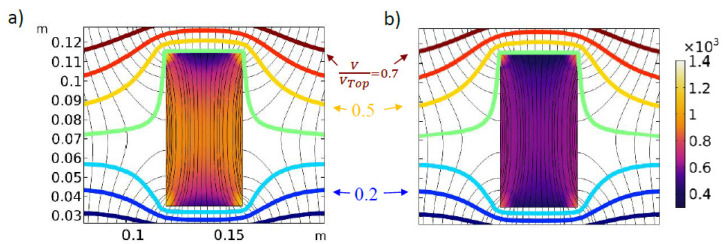
Simulated electric field values during heating of sample 4C, at middle *y-*dimension, 300 W RF nominal power, after (**a**) 1 min and (**b**) 4 min heating. Colored surface: electric field norm (V m^−1^); colored level curves: ratio of electric potential to maximum electric potential; black lines correspond to electric field lines.

**Figure 8:**
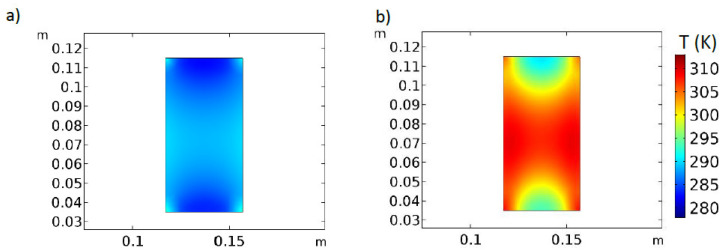
Simulated temperature distribution (K) during heating of sample 4C, at middle *y-* dimension, 300 W RF nominal power, after (**a**) 1 min and (**b**) 4 min heating.

As was shown, the distance between the sample and electrodes influences the thermal response of the sample processed in a RF oven. In the experiments, the distance to the bottom electrode remains constant for the complete set of sample configurations tested. On the contrary, when a second layer of cubes is employed, the distance to the top electrode diminishes significantly. This effect was analyzed through numerical analysis. Figure 7 and Figure 8 show the electric behavior and the sample temperature profile in the middle of y-dimension after 1 min and 4 min RF heating. As can be seen, the electric field largely varies with position and time. At the sample center, the initial norm of the electric field was 1065.7 V m^−1^, while at 4 min it was 595.95 V m^−1^ (results not shown). Electric field variations, together with dielectric properties variations, affect the heat absorbed by the sample. 

After 1 min heating (Figure 7a), the simulated volume average electromagnetic heat source (Equation (23)) was 524 kW m^−3^, whereas after 4 min heating (Figure 7b), it was 369 kW m^−3^. This variation represents a 29.6% reduction in the average heat source. Figure 8 shows the temperature profile in the sample, which follows the electric field distribution depicted in Figure 7, with higher temperature values in the corners and the sample center. The simulated electric field deformations due to the sample are like the ones reported by [6,20], confirming that the uneven distribution of the electric field is responsible of the lack of uniformity in the sample temperature. In this sense, Tiwari et al. [20] found that power uniformity could be improved when the sample projected area is similar to the electrode size, since less electric field distortion is verified.

### 3.3. Energy Efficiency

In order to analyze the heating efficiency of the RF oven tested in this work, Table 7 details experimental and simulated values of energy consumption and efficiency, for the whole set of studied cases. The experimental values of energy consumption (i.e., energy absorbed by the sample) were calculated as the numerator of Equation (5), and the simulated ones by Equation (18). Considering the nominal energy provided in each condition, efficiency was evaluated by using Equations (5) and (19) for experimental and simulated values, respectively. Despite the differences between experimental and simulated values, both sets are highly correlated (r ≅ 0.9991).

The results show that efficiency is higher when the spatial configuration has more than one layer, with a smaller gap between the sample and the top electrode (2B, 4C, 8B, 8C, 12, 16), in coincidence with the higher temperature increase and worse temperature uniformity. Notwithstanding, the bigger samples (8B, 8C, 12, 16) the higher the efficiencies, denoting a better exploitation of the provided energy. 

The tendencies of experimental and simulated efficiency values presented in this work agree with simulated values obtained in a similar system [3], despite the differences in the operating characteristics (27.12 vs. 40.68 MHz) of the cavity and sample configurations. The bigger the load and the smaller the gap between the sample and the top electrode, the higher the heating efficiency. In Uyar et al. [3], the predicted heating efficiency was near zero for a hypothetical small sample that was 1% of the maximum simulated sample size (0.398 L). Efficiency increased as the sample size increased, reaching values near 40–50% for the maximum simulated sample size. Additionally, for the same mass, the spatial configuration with the smallest gap between the top electrode and the sample had the highest efficiencies. 

In general, the energy efficiency observed in bigger samples is higher than the efficiency observed in traditional convective ovens: efficiency from 7.7 to 18.3% was measured in meat cooking [33] and from 6 to 13% in bakery products [34,35].

## 4. Conclusions

In the RF heating of chicken meat at 40.68 MHz, the geometrical factors, such as the spatial configuration of the sample and the gap between top electrode and samples, play a major role in heating rate, temperature uniformity, power absorption and energy efficiency. A decrease in the electrode-sample gap improves heating rates and energy efficiency, but a less uniform temperature distribution is attained. Therefore, the possibility of changing the electrode gap is a qualifying point in the design of RF assisted heating systems. 

Numerical results agree with experimental measurements and show the same general tendencies. 

Both measured and simulated energy efficiencies were close to 50% for the maximum loads, substantially higher than efficiencies obtained in traditional ovens. Notwithstanding, the lack of temperature uniformity is still an issue that deserves a major research effort. The developed mathematical model, validated by comparing the average temperature evolution in the samples, can be extended to different food geometries and RF systems, so that the presented methodology and the digital tool developed in this work can be used in RF heating system design.

## Figures and Tables

**Table 1:** Spatial configuration and main characteristics of samples employed in each experiment.

Sample Code	Spatial Configuration (N Cubes per Width) × (N of Cubes per Length) × (N of Cubes along the Height)	Sample Size (Width × Length × Height) (cm)	Projected Area (cm^2^)	Mass (kg)	Heating Time *t_h_* (min)
2A	1 × 2 × 1	4 × 8 × 4	32	0.12	2
2B	1 × 1 × 2	4 × 4 × 8	16	0.12	2
4A	1 × 4 × 1	4 × 16 × 4	64	0.24	4
4B	2 × 2 × 1	8 × 8 × 4	64	0.24	4
4C	1 × 2 × 2	4 × 8 × 8	32	0.24	4
8A	2 × 4 × 1	8 × 16 × 4	128	0.48	8
8B	2 × 2 × 2	8 × 8 × 8	64	0.48	8
8C	1 × 4 × 2	4 × 16 × 8	64	0.48	8
12	2 × 3 × 2	8 × 12 × 8	96	0.72	12
16	2 × 4 × 2	8 × 16 × 8	128	0.96	16

**Table 2:** Effect of meshing refinement on temperature increase and electromagnetic heat source for sample 16 (16 cubes) for 300 W RF nominal power.

Mesh Refinement	N° of Elements	Final Average Values		Simulation Time (s)
		$T_{Inc,sim}\;(K)$	${\dot{Q}}_{Ave}\;(W\;m^{-3})$	
Extremely coarse	240	51.9	163,930	23
Extra coarse	597	47.9	155,420	24
Coarser	1442	43.2	137,150	33
Coarse	2521	42.1	133,630	43
Normal	7912	40.3	127,470	108
Fine	14258	39.8	125,470	179
Finer	48326	39.2	123,390	593
Extra fine	187631	38.7	121,560	2735

**Table 3:** Average experimental heating rate calculated according to Equation (1), for two RF nominal powers.

Sample Code	Heating Rate *HR* (K s^−1^)	
	225 W	300 W
2A	0.045	0.052
2B	0.133	0.217
4A	0.029	0.041
4B	0.031	0.034
4C	0.101	0.126
8A	0.018	0.025
8B	0.057	0.073
8C	0.051	0.073
12	0.036	0.050
16	0.032	0.049

**Table 4:** Prediction accuracy of the mathematical model.

Sample Code	225 W Power		300 W Power	
	AARD (%)	AAD (K)	AARD (%)	AAD (K)
2A	0.25	0.70	0.05	0.15
2B	0.14	0.41	0.21	0.63
4A	0.06	0.18	0.19	0.53
4B	0.33	0.94	0.14	0.40
4C	0.35	1.03	0.17	0.51
8A	0.04	0.11	0.09	0.27
8B	0.67	1.98	0.11	0.33
8C	0.23	0.66	0.25	0.74
12	0.51	1.50	0.34	1.03
16	0.53	1.58	0.54	1.66
Average	0.31	0.91	0.21	0.63

**Table 5:** Experimental and simulated values of average temperature increase (T-T_0_), calculated at the final heating time, for two RF nominal powers.

Sample Code	*T_Inc,exp_* (K) 225 W	*T_Inc,sim_* (K) 225 W	*T_Inc,exp_* (K) 300 W	*T_Inc,sim_* (K) 300 W
2A	5.99	4.49	6.22	5.96
2B	16.01	15.35	26.02	24.78
4A	6.96	6.86	9.75	9.02
4B	7.36	5.91	8.18	7.76
4C	24.17	23.10	30.21	29.78
8A	8.72	8.68	11.95	11.28
8B	27.46	24.87	35.16	34.95
8C	24.58	24.37	35.11	34.24
12	25.60	24.80	35.81	34.63
16	30.52	28.23	42.99	39.16

**Table 6:** Experimental and simulated values of average temperature uniformity, Equations (4) and (18) respectively, for two RF nominal powers.

Sample Code	*T_U,exp_* (K) 225 W	*T_U,sim_* (K) 225 W	*T_U,exp_* (K) 300 W	*T_U,sim_* (K) 300W
2A	1.5	1.9	2.0	2.1
2B	3.7	3.5	5.7	4.9
4A	3.5	2.5	3.5	2.8
4B	3.4	2.7	3.9	3.0
4C	5.5	5.4	6.3	6.2
8A	4.1	3.6	4.5	4.0
8B	6.4	5.8	7.6	7.5
8C	6.2	5.9	7.3	7.3
12	6.6	6.0	7.9	7.6
16	6.9	6.6	7.9	8.3

**Table 7:** Experimental and simulated values of absorbed energy and efficiency, for two RF nominal powers.

Sample Code	Nominal Supplied Energy, *t_h_ × NP* (kJ)	Absorbed Energy (kJ)		Efficiency (%)	
		Q_Exp_	Q_Sim_	η_Exp_	η_Sim_
225 W power					
2A	27	2.8	2.2	10.3%	8.1%
2B	27	7.5	7.5	27.6%	27.6%
4A	54	6.5	6.7	12.0%	12.4%
4B	54	6.9	5.7	12.7%	10.6%
4C	54	22.5	22.4	41.7%	41.5%
8A	108	16.3	16.9	15.1%	15.6%
8B	108	51.2	48.3	47.4%	44.7%
8C	108	45.8	47.3	42.4%	43.8%
12	162	71.6	72.2	44.2%	44.6%
16	216	113.8	109.6	52.7%	50.7%
300 W power					
2A	36	2.9	2.9	8.1%	8.1%
2B	36	12.1	12	33.7%	33.3%
4A	72	9.1	8.8	12.6%	12.2%
4B	72	7.6	7.5	10.6%	10.4%
4C	72	28.2	28.9	39.1%	40.1%
8A	144	22.3	21.9	15.5%	15.2%
8B	144	65.6	67.8	45.5%	47.1%
8C	144	65.5	66.4	45.5%	46.1%
12	216	100.2	100.8	46.4%	46.7%
16	288	160.3	151.9	55.7%	52.7%

## Data Availability

The data presented in this study are available in this article.
